# Influenza vaccination rates among healthcare workers: a systematic review and meta-analysis investigating influencing factors

**DOI:** 10.3389/fpubh.2023.1295464

**Published:** 2023-11-06

**Authors:** Jingchun Fan, Shijie Xu, Yijun Liu, Xiaoting Ma, Juan Cao, Chunling Fan, Shisan Bao

**Affiliations:** ^1^Center for Laboratory and Simulation Training, School of Public Health, Centre for Evidence-Based Medicine, Gansu University of Chinese Medicine, Lanzhou, China; ^2^School of Public Health, Gansu University of Chinese Medicine, Lanzhou, China; ^3^Social and Historical Sciences, University College London, London, United Kingdom; ^4^School of Nursing, Gansu University of Chinese Medicine, Lanzhou, China; ^5^Department of Public Health, Affiliated Hospital of Gansu University of Chinese Medicine, Lanzhou, China; ^6^Department of Pharmacy, Gansu Provincial Cancer Hospital, Gansu Provincial Academic Institute for Medical Research, Lanzhou, China

**Keywords:** influenza vaccine, vaccination rate, healthcare workers, influencing factors, meta-analysis

## Abstract

**Introduction:**

Healthcare workers risk of exposure to the influenza virus in their work, is a high-risk group for flu infections. Thus WHO recommends prioritizing flu vaccination for them–an approach adopted by >40 countries and/or regions worldwide.

**Methods:**

Cross-sectional studies on influenza vaccination rates among healthcare workers were collected from PubMed, EMBASE, CNKI, and CBM databases from inception to February 26, 2023. Influenza vaccination rates and relevant data for multiple logistic regression analysis, such as odds ratios (OR) and 95% confidence intervals (CI), were extracted.

**Results:**

A total of 92 studies comprising 125 vaccination data points from 26 countries were included in the analysis. The meta-analysis revealed that the overall vaccination rate among healthcare workers was 41.7%. Further analysis indicated that the vaccination rate was 46.9% or 35.6% in low income or high income countries. Vaccination rates in the Americas, the Middle East, Oceania, Europe, Asia, and Africa were 67.1, 51.3, 48.7, 42.5, 28.5, and 6.5%, respectively. Influencing factors were age, length of service, education, department, occupation, awareness of the risk of influenza, and/or vaccines.

**Conclusion:**

The global influenza vaccination rate among healthcare workers is low, and comprehensive measures are needed to promote influenza vaccination among this population.

**Systematic review registration:**

www.inplysy.com, identifier: 202350051.

## Introduction

The World Health Organization (WHO) reports that the flu causes 3 to 5 million severe cases and contributes to 290,000 to 650,000 respiratory disease-related deaths globally p.a (1). Thus flu imposes a substantial impact on both public health and the economy, i.e., the flu resulted in 145,000 deaths, 9.459 million hospitalizations, and 81.536 million hospitalization days due to lower respiratory tract infections (LRTIs), with the flu accounting for 11.5% of LRTI cases in 2017 (2). This aligns with that indirect costs accounted for 88% of the overall economic burden of flu in the 18–64 age group, with 75% of direct costs attributed to hospitalization. Additionally, the costs associated with flu increase with age and the presence of underlying diseases within the 18–64 age group (3).

Annual flu vaccination is widely recognized as an effective preventive measure against the flu. Evidence from a systematic review of randomized controlled trials indicates that inactivated flu vaccines administered to healthy adults can prevent 59% of laboratory-confirmed flu cases, furthermore, when the vaccine strains closely match the circulating flu virus strains, it has been shown to reduce the incidence of influenza-like illness (ILI) by 42% (4).

Healthcare workers face a significant risk of exposure to the flu virus in their daily work, making them a high-risk group for flu infections. A meta-analysis revealed that the incidence of lab-confirmed flu among non-vaccinated healthcare workers was 18.7%, which is 3.4 times higher than the rate observed in healthy adults (5). When healthcare workers contract the flu, it can lead to heightened absenteeism, causing disruptions in medical services and a greater risk of hospital-acquired infections. Furthermore, continuing to work while infected can potentially facilitate the transmission of the flu to other individuals, particularly their family members.

Influenza vaccination is the most significant prevention measure. Recognizing the importance of protecting healthcare workers and preventing the spread of flu, WHO recommends that healthcare workers be given priority for flu vaccination. This recommendation has been adopted by over 40 countries and regions worldwide. However, vaccination coverage exhibited significant variations from one country to another (6), and in some instances, it was notably low (7). In this current systematic review, our objective is to examine the influenza vaccination rates among healthcare workers and the factors that impact their adherence to flu vaccination.

## Methods

### Study type

This meta-analysis included cross-sectional studies that reported the seasonal influenza vaccination rate among healthcare workers.

### Study population

The study population consisted of healthcare workers and healthcare professionals directly involved in providing health services globally.

### Outcome measures

The primary outcome measure of interest was the seasonal influenza vaccination rate, which was defined as the percentage of vaccinated individuals among the total survey population.

### Inclusion criteria

To be included in this meta-analysis, studies had to meet the following criteria:

1. Studies reporting the seasonal influenza vaccination rate among healthcare workers and/or its influencing factors.
2. The study population included healthcare workers and healthcare professionals directly involved in providing health services globally.
3. Studies provided specific information on sample size, vaccination rates, and the number of vaccinated individuals within a given year.
4. Studies were published in either Chinese or English.
5. The study design was cross-sectional.

### Exclusion criteria

The following criteria were used to exclude studies from this meta-analysis:

1. Studies reporting on types of influenza vaccines other than seasonal influenza vaccines.
2. Studies that did not report key data such as sample size, vaccination rates, and the number of vaccinated individuals, or studies that did not specify the vaccination year or only reported combined vaccination rates for multiple years.
3. Studies that focused solely on healthcare institutions or the overall population of a country, without specific data on healthcare workers.
4. Duplicate publications, where the same study was published in multiple sources.
5. Studies with logical errors or inconsistencies in the reported data.

### Literature search strategy

Computer-based searches were performed in multiple databases, including PubMed, EMBASE, CNKI, CBM, Wanfang, and VIP. The search aimed to identify cross-sectional studies that reported the seasonal influenza vaccination rate among healthcare workers. The search was conducted from the inception of each database up to February 26, 2023. The search strategy utilized a combination of subject terms and free-text terms, Search, terms like “Influenza Vaccine*,” “Flu Vaccine*,” “Influenza Virus Vaccine*,” “Universal Influenza Vaccine*,” “Universal Flu Vaccine*,” “Immunization Coverage*” and “Vaccination Coverage*” were utilized. This comprehensive search strategy was designed to capture relevant studies and gather a wide range of literature on the seasonal influenza vaccination rate among healthcare workers (Supplementary Table S1).

### Literature screening and data extraction

The identified literature was imported into Endnote literature management software, and duplicate records were removed. Two researchers independently screened the literature and performed data extraction. In cases of discrepancies, a third senior researcher was consulted for discussion and to reach a consensus. Initially, the title and abstract of each article were reviewed to exclude obviously irrelevant studies. Subsequently, the full text of the remaining articles was thoroughly examined to determine their eligibility for inclusion in the meta-analysis.

Data extraction encompassed various key aspects, including the first author’s name, publication year, survey region, sampling location, study population, vaccination time, sample size, number of vaccinated individuals, and relevant data from multiple logistic regression analysis, such as odds ratios (ORs), 95% confidence intervals (CIs), and reference objects. This rigorous screening and data extraction process ensured that relevant and reliable information was obtained from the selected studies for further analysis.

### Evaluation of bias risk in included studies

To assess the methodological quality of the included cross-sectional studies, a checklist was developed based on recommended guidelines. This checklist incorporated items from the cross-sectional study quality evaluation tool endorsed by the Agency for Healthcare Research and Quality (AHRQ) and the JBI Analytic Cross-Sectional Study Quality Evaluation Scale.

The checklist consisted of nine key items aimed at evaluating the potential biases in the included studies. These items included:

1. Clearly stating the source of data (e.g., survey, literature review).
2. Clearly defining the inclusion criteria for the study population.
3. Providing detailed descriptions of the study population and study site.
4. Offering an explanation for the exclusion of certain study subjects from the analysis.
5. Summarizing the patient response rate and data collection completeness.
6. Explaining how missing data was handled during the analysis if the research data was incomplete or had missing values.
7. Describe how confounding was assessed and/or controlled.
8. Whether to use effective and credible methods to measure outcome indicators.
9. Whether the data analysis method is appropriate.

By systematically assessing these aspects, the checklist enabled a comprehensive evaluation of the methodological quality of the cross-sectional studies. This evaluation helped to identify any potential biases that may have influenced the study results and ensured the reliability of the findings.

### Data analysis

The data extraction and analysis were performed using Excel 2016 and STATA 12.0 software. To assess publication bias, Egger’s test and funnel plot were utilized. A significance level of 0.05 or 0.01 was considered statistically significant. Given the anticipated heterogeneity, a random-effects model was employed for the analysis. Sensitivity analysis was conducted to assess the robustness and reliability of the overall vaccination rate estimate. Additionally, subgroup analysis was performed to explore potential sources of heterogeneity.

For the analysis of vaccination rates, the formula used was as follows:

Influenza vaccine vaccination rate = number of vaccinators / sample size.

The standard error of the rate was calculated using the formula:

Standard error of rate = sqrt (rate × (1-rate) / sample size).

When adequate data were available from the included articles, the random effects model was utilized to estimate the odds ratios (OR) of the influencing factors. This approach allowed for a comprehensive assessment of the relationship between the influencing factors and the vaccination rates.

These analytical methods were employed to ensure a comprehensive evaluation of the data and to derive reliable and robust outcomes from the study. By utilizing these methods, we aimed to provide accurate and valid insights into the influencing factors of influenza vaccination rates among healthcare workers.

## Results

### During the literature screening process

**Figure 1:**
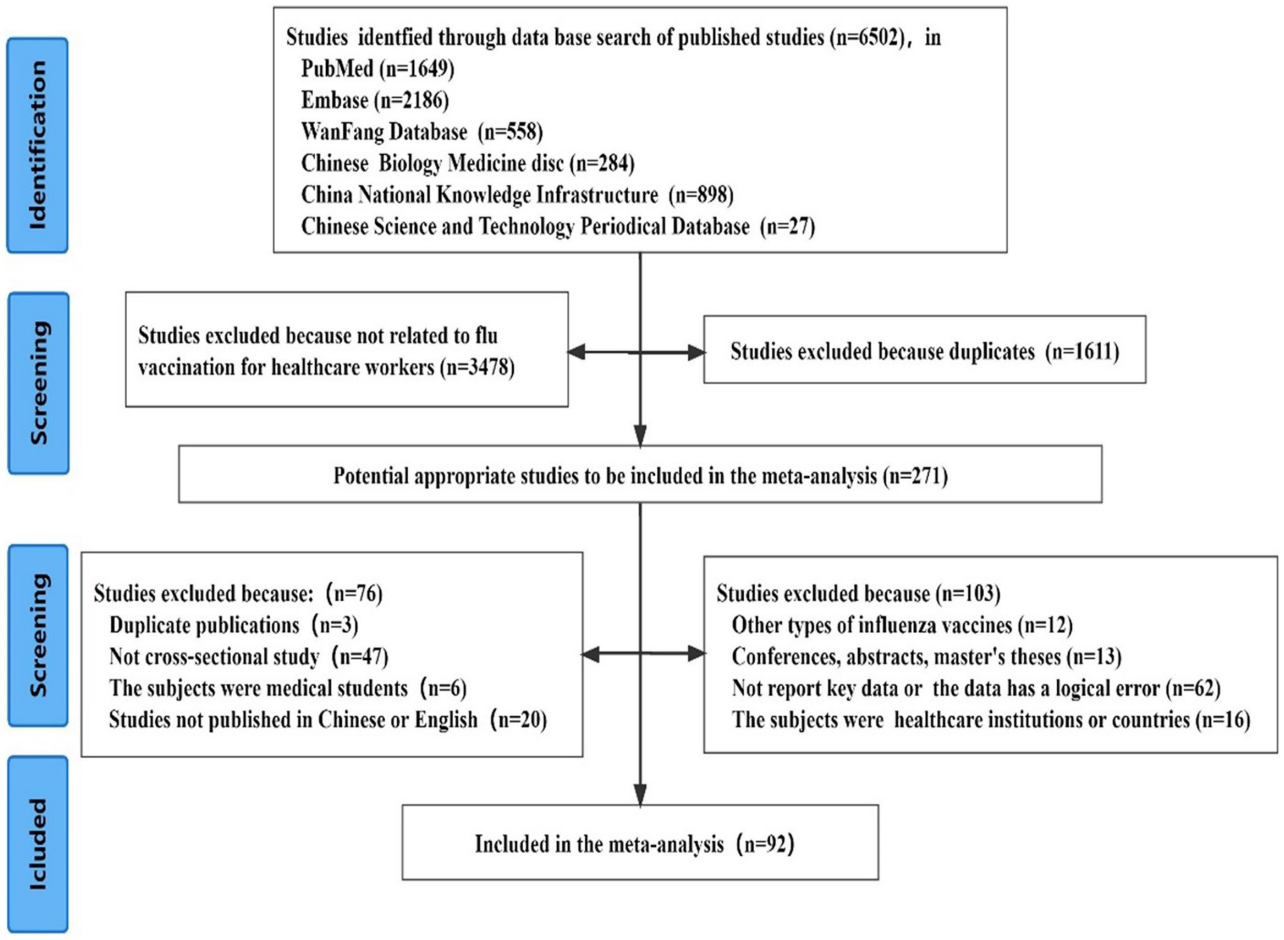
The detailed process and results of the literature screening.

A comprehensive search of relevant articles yielded a total of 6,502 records. Following the screening process, 92 cross-sectional studies were considered eligible for inclusion in the analysis. The detailed process and results of the literature screening are presented in Figure 1. These 92 studies encompassed 125 data points on influenza vaccination, with sample sizes ranging from 106 to 8,975 participants. The reported vaccination rates varied between 3.1 and 99.6%. The studies were conducted in 26 countries across Asia, Europe, the Americas, Africa, Oceania, and the Middle East, providing a diverse geographical representation.

It is summarized that the key characteristics of the included studies, including their basic information and vaccination data (Table 1). The evaluation of literature quality resulted in an average score of 7.86 points. Among the included articles, one was rated as low-quality, 30 as medium-quality, and 61 as high-quality studies.

**Table 1:** Basic information of literatures of included studies.

Study	Sampling location	Population	Vaccination time	Study region	Sample size	Vaccination population	Quality score
Sheng et al. (8)	Internet survey	Nurses	2017	Mainland China	773	31	8
Liu et al. (9)	Community health centers	All HCWs	2018	Mainland China	1,359	424	9
Wang et al. (10)	Hospital	All HCWs	2012	Mainland China	569	171	9
Gao et al. (11)	Hospital	All HCWs	2013	Mainland China	369	51	8
Liu et al. (12)	Hospital	Nurses	2018	Mainland China	299	68	9
Yang and Chen (13)	Hospital	Nurses	2013	Mainland China	650	284	9
Gan et al. (14)	Community health centers	All HCWs	2018	Mainland China	106	24	9
Wang (15)	Hospital	All HCWs	2007	Mainland China	199	15	6
Bu et al. (16)	Hospital	All HCWs	2012	Mainland China	1,521	98	8
Yang et al. (17)	Hospital	All HCWs	2016	Mainland China	1941	107	9
Wang et al. (18)	Internet survey	Nurses	2017	Mainland China	510	16	8
Zhang et al. (19)	Hospital	All HCWs	2017	Mainland China	943	131	9
Kong et al. (20)	Hospital/Community health centers /CDC	All HCWs	2019	Mainland China	8,975	2,241	9
Ma et al. (21)	Hospital	All HCWs	2017	Mainland China	3,260	226	8
Gan et al. (22)	Influenza sentinel surveillance hospital/Hospital	All HCWs	2018	Mainland China	1,412	237	8
James et al. (23)	Hospital	All HCWs	2016	Sierra Leone	706	46	8
Liu et al. (24)	Internet survey	All HCWs	2018	Mainland China	4,078	472	9
Hosamirudsari et al. (25)	Hospital	All HCWs	2015	Iran	378	218	7
Alhammadi et al. (26)	Hamad Medical Corporation	All HCWs	2013	Qatar	230	151	9
Boey et al. (27)	Hospital/Nursing homes	All HCWs	2014	Belgium	450	334	9
Barbadoro et al. (28)	National Health Surveys.	All HCWs	2012	Italy	5,336	1,110	6
Wong et al. (29)	Hospital	Nurses	2017	Hong Kong	708	309	5
Kyaw et al. (30)	Hospital	All HCWs	2015	Singapore	3,873	3,191	9
Rabensteiner et al. (31)	Health Service	All HCWs	2015	Italy	4,091	425	9
Garcell and Ramirez (32)	Hospital	All HCWs	2012	Qatar	325	231	6
Esposito et al. (33)	University Hospital	All HCWs	2006	Italy	2,143	432	9
Hudu et al. (34)	Hospital	All HCWs	2013	Malaysia	527	271	7
Costantino et al. (35)	University	Medical residents	2011	Italy	2,506	299	9
Jimenez-Garcia et al. (36)	National Health Surveys.	All HCWs	2003	Spain	518	102	8
Von Perbandt et al. (37)	Hospital	All HCWs	2014	Switzerland	200	30	8
Haridi et al. (38)	Medical City	All HCWs	2014	Saudi Arabia	447	394	9
Sočan et al. (39)	Slovenian Medical Chamber	Physicians and dentists	2009	Slovenia	1718	890	8
Domínguez et al. (40)	Healthy primary facilities	All HCWs	2011	Spain	1749	887	9
Rehmani and Memon (41)	Hospital	All HCWs	2008	Saudi Arabia	512	176	9
Kan et al. (42)	Hospital	Nurses	2011	Mainland China	895	295	9
Kent et al. (43)	Public Health Directorates	All HCWs	2007	America	1,203	871	9
Hagemeister et al. (44)	Hospital	All HCWs	2012	Germany	675	286	7
Castilla et al. (45)	Hospital	All HCWs	2008	Spain	1965	1,203	8
Ball et al. (46)	National opt-in Internet panels	All HCWs	2012	America	1944	1,400	7
Black et al. (47)	National opt-in Internet panels	All HCWs	2013	America	1882	1,415	7
Black et al. (48)	National opt-in Internet panels	All HCWs	2014	America	1914	1,480	7
Black et al. (49)	National opt-in Internet panels	All HCWs	2015	America	2,258	1784	7
Black et al. (50)	National opt-in Internet panels	All HCWs	2016	America	2,438	1916	7
Black et al. (51)	National opt-in Internet panels	All HCWs	2017	America	2,265	1776	7
CDC (52)	National opt-in Internet panels	All HCWs	2010	America	1931	1,226	7
Ball at al. 2012 (53)	National opt-in Internet panels	All HCWs	2011	America	2,348	1,571	7
Tanguy et al. (54)	Hospital	All HCWs	2009	France	532	119	5
Amodio et al. (55)	University Hospital	Medical residents	2009	Italy	202	44	8
Hakim et al. (56)	Hospital	All HCWs	2018	Egypt	3,534	1,087	9
Hussain et al. (57)	Hospital	All HCWs	2013	Canada	896	654	7
Tagajdid et al. (58)	Hospital	All HCWs	2011	Morocco	721	122	6
Dorribo et al. (59)	University Hospital	All HCWs	2009	Switzerland	472	245	9
Bazán et al. (60)	Hospital/Health centers	All HCWs	2010	Peru	672	544	9
Yi et al. (61)	Internet survey	All HCWs	2019	Mainland China	4,366	2,927	8
Sánchez-Payá et al. (62)	University Hospital	All HCWs	2010	Spain	3,126	762	8
Yu et al. (63)	Internet survey	Nurses	2017	Mainland China	4,153	257	8
Groenewold et al. (64)	Nursing homes	Nurses	2004	America	2,873	107	6
Hajiabdolbaghi et al. (65)	Hospital	All HCWs	2019	Iran	637	189	5
Dubnov et al. (66)	Hospital	All HCWs	2004	Israel	256	42	7
Buxmann et al. (67)	Hospital	All HCWs	2016	Germany	124	49	9
Khazaeipour et al. (68)	University Hospital	All HCWs	2008	Iran	139	93	7
Lu and Euler (69)	National Health Surveys.	All HCWs	2006	America	484	226	6
Domínguez et al. (70)	Hospital	All HCWs	2011	Spain	1749	886	8
Toledo et al. (71)	community health centers	Pharmacists	2013	Spain	463	116	9
Loulergue et al. (72)	Medical departments	All HCWs	2006	France	395	204	8
Madewell et al. (73)	Hospital	All HCWs	2018	America	706	393	8
Harrison et al. (74)	Hospital	Nurses	2013	Austria	107	45	8
Petek and Kamnik-Jug (75)	Primary care centers	All HCWs	2014	Slovenia	250	30	9
Murray and Skull (76)	Hospital	All HCWs	1999	Australia	269	131	7
Mojamamy et al. (77)	Primary care centers	All HCWs	2015	Saudi Arabia	368	320	7
Vírseda et al. (78)	University Hospital	All HCWs	2009	Spain	527	262	8
Amani et al. (79)	Hospital/community health centers	All HCWs	2019	Egypt	980	131	9
Hämäläinen et al. (80)	University Hospital	All HCWs	2015	Finland	985	586	7
Khazaeipour et al. (81)	University Hospital	All HCWs	2008	Iran	139	93	9
Jiang et al. (82)	Hospital	All HCWs	2019	Mainland China	2,974	713	8
Fan et al. (83)	Hospital	All HCWs	2019	Mainland China	6,654	1,037	7
Yan et al. (84)	Hospital	All HCWs	2019	Mainland China	1,332	614	7
Li et al. (85)	Hospital	All HCWs	2020	Mainland China	4,135	2,460	9
Zhang et al. (86)	Hospital	All HCWs	2019	Mainland China	775	255	9
Wu et al. (87)	Hospital	All HCWs	2018	Mainland China	3,507	413	8
Lv et al. (88)	Community health centers	All HCWs	2018	Mainland China	1,483	216	8
Fan et al. (89)	Hospital	All HCWs	2020	Mainland China	769	670	9
Lei et al. (90)	Influenza sentinel surveillance Hospital	All HCWs	2020	Mainland China	1854	419	9
Ma et al. (91)	Internet survey	All HCWs	2021	Mainland China	1,697	600	9
Papageorgiou et al. (92)	Health care services institutions	All HCWs	2019	Cyprus	962	306	8
Ajejas Bazán et al. (93)	Public Health Directorates	All HCWs	2020	Spain	832	590	8
Bertoni et al. (94)	Cancer research institute	All HCWs	2020	Italy	579	334	8
Marinos et al. (95)	Athens Medical Association	All HCWs	2020	Greece	1993	1,523	7
Shi et al. (96)	Hospital/Community health centers	All HCWs	2020	Mainland China	2,192	868	8
Jędrzejek and Mastalerz-Miga (97),	Hospital	All HCWs	2019	Poland	165	101	8
Costantino et al. (98)	Community health centers	Pharmacists	2020	Italy	1,450	841	7
Ogliastro et al. (99)	University Hospital	All HCWs	2021	Italy	4,753	1,423	4

### Influenza vaccination rate and subgroup analysis

The meta-analysis included a total of 92 cross-sectional studies, and a random effects model was employed. The analysis revealed that the global influenza vaccination rate among healthcare workers was 41.7% (95% CI [35.7, 47.7%)]. However, it is noted that significant heterogeneity was observed among the studies (*I^2^* = 99.9%, *p* < 0.001). To further explore the sources of heterogeneity, subgroup analyzes were conducted based on the country’s level of development, geographic region, and time of vaccination.

The countries included in the analysis were categorized as low income or high income according to their economic levels. It was revealed that the influenza vaccination rate among healthcare workers in developed or developing countries was 46.9% or 35.6%. Furthermore, the study regions were classified into Asia, Europe, America, Africa, Oceania, and the Middle East based on their geographical locations. Subgroup analysis revealed that America had the highest vaccination rate at 67.1%, followed by the Middle East, Oceania, Europe, and Asia with rates of 51.3, 48.7, 42.5, and 28.5%, respectively. Africa had the lowest vaccination rate at 6.5%. The study periods were divided based on the occurrence of the H1N1 influenza pandemic (March 2009 to August 2010) and the COVID-19 epidemic (from the end of December 2019). The vaccination rates were separately analyzed for different periods: before 2009, 2009–2012, 2013–2016, 2017–2019, and 2020-present. The subgroup analysis showed that the highest vaccination rate was observed since 2020 at 52.8%, followed by the period of 2009–2012 at 46.7%, 2013–2016 at 46.5%, before 2009 at 39.4%, and the lowest rate was during 2017–2019 at 31.4%.

Despite the subgroup analysis, there remained high heterogeneity in the vaccination rates within each subgroup, indicating that the level of economic development, geographical location, and different vaccination periods were not the primary sources of heterogeneity. The detailed results of the subgroup analysis can be found in Table 2.

**Table 2:** Influenza vaccination rate of HCWs in different groups.

Groups	Reference(n)	Test of heterogeneity result			Meta-analysis results	
		*P*	*I^2^*(%)	Effect model	Rate (%)	95% CI
Economic development levels						
Developing country	67	<0.001	99.9	Random	46.9	(38.0, 55.9%)
Developed country	58	<0.001	99.8	Random	35.6	(30.1, 41.1%)
Geographic region						
Asia	45	<0.001	99.8	Random	28.5	(23.2, 33.8%)
Europe	45	<0.001	99.9	Random	42.5	(31.2, 53.8%)
America	17	<0.001	99.9	Random	67.1	(48.9, 85.4%)
Africa	1	–	–	Random	6.5	(4.7, 8.3%)
Oceania	1	–	–	Random	48.7	(42.7, 54.7%)
Middle East	16	<0.001	99.6	Random	51.3	(38.1, 64.5%)
Vaccination time						
~2008	13	<0.001	99.8	Random	39.4	(21.9, 56.8%)
2009–2012	28	<0.001	99.8	Random	46.7	(37.9, 55.6%)
2013–2016	33	<0.001	99.8	Random	46.5	(35.8, 57.2%)
2017–2019	39	<0.001	100.0	Random	31.4	(18.5, 44.3%)
2020~	12	<0.001	99.7	Random	52.8	(41.9, 63.8%)
Total	125	<0.001	99.9	Random	41.7	(35.7, 47.7%)

### Publication bias test

**Figure 2:**
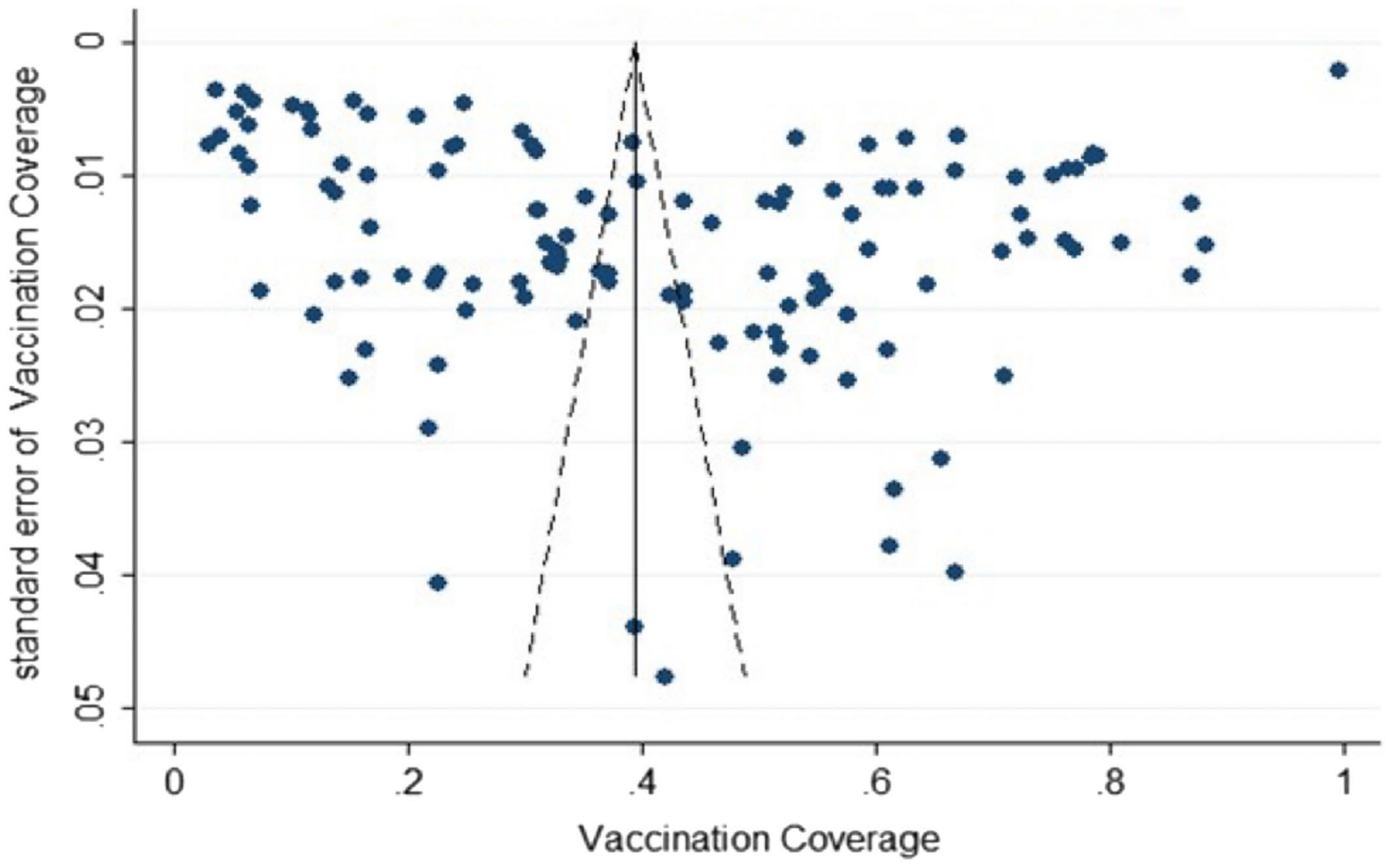
Funnel plot with pseudo 95% confidence limits.

A funnel plot was generated using the 125 vaccination rate data included in the study (Figure 2), which showed that the scatter was relatively dispersed and roughly symmetrical. The Egger’s test confirmed that there was no significant publication bias in the studies (*t* = −0.33, *p* = 0.741), indicating that this study had low publication bias.

### Sensitivity analysis

A sensitivity analysis was performed by systematically excluding individual studies from the meta-analysis. The results indicated that the effect size remained consistent, ranging from 41 to 43%, even when each study was removed, suggesting that the meta-analysis findings were robust and stable (Supplementary Table S2).

### Factors influencing influenza vaccination

A total of 32 factors were identified from the included studies that significantly influenced healthcare workers’ uptake of influenza vaccine. Several factors played a significant role in influencing vaccination uptake among healthcare workers, including age, length of employment, education level, department of work, occupation, presence of chronic diseases, perception of being at risk of infection, belief in vaccine effectiveness, willingness to receive vaccination, recommendation of influenza vaccine to patients, previous COVID-19 vaccination, participation in influenza or influenza vaccine training and health education, and knowledge of vaccination timing.

Compared with the younger age group, the middle-aged and older adult groups were more likely to receive the vaccine. Healthcare workers with more than 10 years of experience were more likely to be vaccinated than those with less than 10 years of experience. Non-clinical staff were more likely to receive the vaccine than clinical staff. Among healthcare workers who had chronic diseases, perceived themselves to be at high risk of infection, believed in the effectiveness of the vaccine, had the willingness to receive the vaccine, recommended the vaccine to patients, had previous COVID-19 vaccination, and had knowledge of vaccination timing, were more likely to receive the influenza vaccine.

Subgroup analysis of influencing factors showed that gender, marital status, professional title, perception of vaccine safety, source of vaccine information, and whether the workplace provided free vaccines may also be factors influencing healthcare workers’ uptake of influenza vaccine. The detailed findings of these significant factors are summarized in Table 3.

**Table 3:** Factors associated with influenza vaccination rates among health care workers.

Factor	Test of heterogeneity result			Meta-analysis results		*P*
	*P*	*I*^2^(%)	Effect model	OR	OR95%CI	
Sex	<0.001	78.5	Random	1.197	(0.987, 1.452)	0.068
Female	<0.001	71.3	Random	0.960	(0.787, 1.171)	0.687
Male	0.001	85.3	Random	1.656	(1.289, 2.127)	<0.001
Age	<0.001	95.1	Random	1.700	(1.600, 1.807)	<0.001
Younger age	0.001	70.6	Random	1.575	(1.104, 2.247)	0.012
Middle-aged	<0.001	91.0	Random	2.278	(1.790, 2.900)	<0.001
Older adult	<0.001	90.5	Random	2.824	(1.669, 4.779)	<0.001
Whole population	0.001	77.6	Random	1.018	(1.002, 1.034)	0.030
Length of service	<0.001	93.7	Random	1.286	(1.179, 1.402)	<0.001
≤10	<0.001	90.6	Random	1.214	(0.888, 1.659)	0.224
11–30	<0.001	81.6	Random	1.397	(1.203, 1.622)	<0.001
>30	<0.001	84.9	Random	1.414	(0.775, 2.582)	0.259
Other	0.373	0.0	Random	1.009	(0.999, 1.018)	0.075
Education level	<0.001	73.1	Random	0.837	(0.723, 0.969)	0.017
College degree or below	<0.001	76.5	Random	0.721	(0.582, 0.895)	0.003
Bachelor degree	0.154	37.9	Random	0.829	(0.666, 1.033)	0.095
Master degree or above	0.005	70.2	Random	1.076	(0.809, 1.431)	0.616
Marital status	0.054	44.6	Random	1.139	(0.976, 1.329)	0.100
Married/Cohabitant	0.027	60.4	Random	1.096	(0.854, 1.406)	0.473
Separated/Divorced	0.922	0.0	Random	1.086	(0.896, 1.318)	0.400
Widowed	0.716	0.0	Random	1.583	(1.162, 2.158)	0.004
Professional title	0.058	41.6	Random	1.123	(0.992, 1.270)	0.066
Associate senior or above	0.015	67.6	Random	1.238	(0.939, 1.633)	0.130
Middle	0.434	0.0	Random	1.139	(1.027, 1.264)	0.014
Primary	0.110	60.8	Random	1.059	(0.581, 1.933)	0.851
No title	0.857	0.0	Random	0.762	(0.481, 1.208)	0.248
Department	<0.001	77.2	Random	1.435	(1.148, 1.794)	0.241
Clinical	<0.001	89.6	Random	1.177	(0.896, 1.546)	0.002
Non-clinical	<0.001	85.2	Random	1.781	(1.243, 2.551)	0.002
Occupation	<0.001	86.7	Random	1.757	(1.503, 2.055)	<0.001
Nursing staff	<0.001	93.1	Random	1.371	(1.006, 1.868)	0.046
Others	0.016	49.3	Random	1.397	(1.160, 1.682)	<0.001
Clinician	<0.001	80.0	Random	2.365	(1.868, 2.993)	<0.001
Hospital level	<0.001	88.9	Random	0.941	(0.660, 1.340)	0.734
Primary	0.002	84.5	Random	1.315	(0.907, 1.907)	0.148
Secondary	0.033	70.6	Random	0.618	(0.376, 1.015)	0.057
Have children at home	0.465	0.0	Random	1.024	(0.907, 1.155)	0.706
Have old people at home	0.047	62.3	Random	1.347	(0.987, 1.838)	0.060
Have chronic medical condition	0.399	4.5	Random	1.707	(1.441, 2.021)	<0.001
They consider themselves to be at high risk of infection	<0.001	87.7	Random	1.981	(1.256, 3.126)	0.003
Think the vaccine is effective	<0.001	87.7	Random	2.101	(1.249, 3.534)	0.005
Whether the vaccine is safe	<0.001	87.1	Random	1.413	(0.921, 2.169)	0.113
Safe	<0.001	90.2	Random	1.619	(1.008, 2.601)	0.046
Unsafe	0.440	0.0	Random	0.741	(0.349, 1.577)	0.437
Support HCWs to receive influenza vaccination	<0.001	95.2	Random	2.279	(0.824, 6.308)	0.113
Worried about vaccine side effects	0.041	76.0	Random	0.693	(0.312, 1.537)	0.367
That vaccines cause the flu	0.074	68.7	Random	0.834	(0.443, 1.570)	0.575
Protect patients	0.011	84.4	Random	2.154	(0.971, 4.778)	0.059
Willing to vaccinate	0.792	0.0	Random	4.104	(2.421, 6.956)	<0.001
Whether to recommend vaccines to patients	<0.001	86.1	Random	2.193	(1.315, 3.658)	0.003
No	0.293	9.5	Random	1.320	(0.877, 1.986)	0.183
Yes	<0.001	86.1	Random	2.739	(1.524, 4.922)	0.001
COVID-19 vaccination	0.001	91.2	Random	5.922	(1.136, 30.876)	0.035
Have participated in flu or flu vaccine training, health promotion	0.003	89.0	Random	0.773	(0.259, 0.309)	0.645
Yes	..	..	Random	1.288	(1.034, 1.604)	0.024
No	..	..	Random	0.420	(0.420, 0.840)	0.014
Sources of information	<0.001	84.5	Random	1.060	(0.814, 1.380)	0.666
People around me	0.099	63.3	Random	1.174	(0.714, 1.928)	0.527
Mass media	<0.001	92.4	Random	0.665	(0.320, 1.382)	0.275
Professional organization or publication	0.245	24.2	Random	1.301	(1.113, 1.520)	0.001
Know the vaccination time	<0.001	85.2	Random	2.224	(1.165, 4.244)	0.015
Know the vaccine priority groups	<0.001	84.7	Random	1.327	(0.857, 2.053)	0.205
Know that the vaccine is the most effective way to prevent flu	<0.001	88.7	Random	1.031	(0.310, 3.432)	0.960
Know that the vaccine is given once a yea	<0.001	88.5	Random	1.028	(0.612, 1.729)	0.916
Work units participate in the influenza sentinel network	0.850	0.0	Random	0.920	(0.698, 1.213)	0.555
Free vaccination at workplace	<0.001	98.9	Random	0.746	(0.317, 1.756)	0.502
Yes	<0.001	98.3	Random	1.533	(0.525, 4.479)	0.435
No	<0.001	99.4	Random	0.644	(0.087, 4.777)	0.667
Unclear	<0.001	94.6	Random	0.279	(0.088, 0.886)	0.030
Have vaccination sites at workplace	<0.001	91.5	Random	1.377	(0.764, 2.480)	0.287
Workplace attitudes toward influenza vaccination of medical staff	<0.001	95.7	Random	0.897	(0.498, 1.617)	0.718
Ask for or support encouragement	<0.001	94.0	Random	1.602	(0.944, 2.717)	0.081
Do not require or encourage	0.725	0.0	Random	0.300	(0.207, 0.435)	<0.001
Unclear	..	..	Random	0.090	(0.038, 0.216)	<0.001

### Reasons for accepting or refusing influenza vaccination

Among the 92 studies included, 47 studies reported on the reasons why healthcare workers chose to get vaccinated against influenza, while 55 studies reported on the reasons for refusing vaccination. The comprehensive data are summarized in Table 4, providing insights into the factors that influenced healthcare workers’ decisions to either receive or decline influenza vaccination.

**Table 4:** Self-reported reasons for accepting or refusing influenza vaccination in healthcare workers.

Reasons for refusing	Reference(n)	Reasons for accepting	Reference(n)
1. The vaccine is considered to have poor or limited preventive effect	43	1. Protect myself	30
2. Concerns about adverse reactions or vaccine quality	38	2. Protect my family, patients, and people around me	26
3. I’m too busy at work to have time	33	3. Worried about spreading it to the people around me	19
4. They are considered to be in good physical condition or have strong immunity and do not need vaccination	23	4. Vaccines are free or cheap	17
5. Think the flu is mild and will not cause serious illness	21	5. The vaccine is considered effective in preventing influenza and its complications	16
6. Vaccines are out-of-pocket or too expensive	20	6. Consider myself at high risk for the flu and its complications	13
7. Vaccinations are inconvenient or lacking	19	7. A work organization or employer requires or performs professional obligations	13
8. There are contraindications to vaccination	16	8. Recommended or influenced by leaders, colleagues, relatives and friends	12
9. Do not know about influenza vaccination and related information	14	9. Vaccination sites are available or readily available in the workplace	11
10. Not considered to be at high risk of catching the flu	14	10. That flu is a serious illness with serious effects	10
11. Adverse reactions after vaccination (e.g., flu-like symptoms, pain at injection site)	12	11. Avoid infection affecting my work	8
12. Not knowing when and where to get flu shots	11	12. It is recommended by government health authorities or the technical guidelines for influenza vaccines	7
13. Forget to vaccinate	11	13. Old age, underlying disease or chronic disease, fear of complications after infection	7
14. Fear of injection	8	14. Believe in the safety of flu vaccines	4
15. It is considered easy to treat with drugs or prevent with hygiene measures or other drugs	8	15. Doctor’s recommendation	3
16. Concerned about the safety of vaccines	7	16. I had the flu last season	3
17. Being pregnant or lactating	7	17. Participate in multidisciplinary campaigns or influenza vaccination campaigns	2
18. Requires annual vaccinations or immunization procedures	5	18. Have a history of influenza vaccination	2
19. Vaccination is not mandatory or recommended by the workplace	5	19. Familiarize with flu vaccination	1
20. Does not believe in or oppose vaccination	5	20. Flu infections take an economic toll	1
21. Personal choice, reduce drug use	4		
22. There is no awareness of getting the flu vaccine	1		
23. Had the flu this year and do not need to get vaccinated	1		

## Discussion

The present study encompasses a broad range of countries, including 26 nations across 7 different regions. The meta-analysis findings indicate a relatively low global influenza vaccination rate among healthcare personnel, estimated at 41.7%. Subgroup analysis reveals a notable disparity between developed and developing countries, with higher vaccination rates observed in the former. Among regional subgroups, the Americas exhibit the highest vaccination rate, followed by the Middle East, Oceania, and Europe, while Africa demonstrates the lowest rate. These results suggest that variations in socio-economic development, vaccine accessibility, cost, healthcare service standards, healthcare personnel’s knowledge regarding influenza and influenza vaccines, as well as disparities in awareness of preventive healthcare and vaccination, contribute to the observed differences in influenza vaccination rates across countries. This is consistent with a previous report, which highlights that while Chinese clinical workers possess extensive knowledge about disease diagnosis and treatment, their understanding of health maintenance and disease prevention is comparatively lacking (22).

Subgroup analysis based on vaccination time reveals that rate is gradually increased over the period of 14 years, suggesting that the H1N1 influenza pandemic in 2009 and the subsequent COVID-19 epidemic have played a role in promoting the seasonal influenza vaccination rate among healthcare personnel, likely due to increased awareness of the contagious nature of these diseases (95, 99). However the influenza vaccination rate gradually declined since 2009 pandemic, which aligns with the decreasing impact of the influenza outbreak. However, the occurrence of the COVID-19 epidemic led to a surge in the influenza vaccination, reaching its highest level. This could be attributed to heightened focus on self-protection during the influenza season, increased awareness of the importance of influenza vaccines, and a general promotion of vaccination practices.

The analysis of influencing factors reveals that several characteristics contribute to the higher likelihood of healthcare personnel receiving influenza vaccinations, including age, tenure, education level, professional designation (clinical doctors compared to nurses), and their inclination to recommend influenza vaccines to patients. These findings are in line with studies conducted in China (21, 22) and Cyprus (92), which similarly indicate that doctors are more likely to be vaccinated compared to nurses. This discrepancy may be due to doctors increased exposure to influenza patients due to their longer experience in the field, resulting in a stronger sense of identification as a high-risk group for influenza infection. Consequently, doctors exhibit heightened attention and awareness regarding influenza-related knowledge and information on influenza vaccines.

A study conducted in Spain focused on healthcare personnel in the armed forces, the proportion of vaccinated individuals increased with age and years of service in the 2016–2017 season, but the vaccination rate among younger/middle-ranking officers actually surpassed that of the older adult, indicating a notable shift in vaccination behavior in the 2019–2020 season (93). Such outcome could be attributed to the evolving health knowledge system, which now places greater emphasis on disease prevention and health maintenance. In another survey conducted among nurses in North-eastern China, showing an inverse correlation between vaccination and flu among nurses, maybe due to lack of knowledge among these nurses regarding influenza vaccines, necessitating further education and awareness campaigns to emphasize the importance of vaccination.

Our present findings offer valuable insights for promoting flu vaccination, particularly among healthcare workers. This may involve strategies such as cost reduction or even the implementation of mandatory vaccination policies for specific high-risk population groups. Furthermore, our current data could serve as a foundation for future studies and investments in healthcare worker well-being. Our data underscores the critical importance of flu vaccination for these healthcare workers, who often find themselves in more vulnerable conditions, serving both the older adult and other high-risk groups. This relevance is further emphasized by the ongoing threat of viral mutation and the persistence of long-term consequences from COVID-19, even though it is no longer classified as a pandemic. Hence, our present data strongly underscores the critical importance of flu vaccination for healthcare workers, especially those in more vulnerable roles, such as caring for the older adult and other high-risk groups. This relevance is further accentuated by the context of the ongoing COVID-19 outbreak, even if it is no longer considered a pandemic. The continuous viral mutation and the lingering presence of long-term COVID-19 complications make this vigilance particularly vital.

In conclusion, the influenza vaccination rate among healthcare workers globally remains low. To address this issue effectively, it is crucial to implement comprehensive measures that promote influenza vaccination among this population, as well as the general public. Efforts should be focused on raising awareness about the importance of vaccination, providing accessible and convenient vaccination services, and enhancing education regarding influenza and its prevention. By implementing these measures, we can strive to improve the influenza vaccination rates among healthcare workers and the wider population, leading to better overall public health outcomes.

## Data availability statement

The datasets presented in this study can be found in online repositories. The names of the repository/repositories and accession number(s) can be found in the article/Supplementary material.

## Author contributions

JF: Conceptualization, Funding acquisition, Supervision, Writing – original draft, Writing – review & editing. SX: Data curation, Formal analysis, Writing – original draft. YL: Data curation, Visualization, Writing – review & editing. XM: Data curation, Formal analysis, Software, Writing – review & editing. JC: Data curation, Formal analysis, Investigation, Writing – review & editing. CF: Conceptualization, Project administration, Validation, Writing – review & editing. SB: Conceptualization, Project administration, Writing – review & editing.
