# Supplementary material for: Influenza vaccination rates among healthcare workers: a systematic review and meta-analysis investigating influencing factors
Source: Front Public Health. 2023 Nov 6;11:1295464. doi: 10.3389/fpubh.2023.1295464 (PMC10657874; doi:10.3389/fpubh.2023.1295464)
Supplement: Supplementary file 1 [file Table_1.docx]

Supplementary Table 1 Search Strategy (PubMed)

| #1 Search | "Influenza Vaccines"[Mesh] |
| --- | --- |
| #2 Search | (((((((((Influenza Vaccine[Title/Abstract])) OR (Influenza Vaccines[Title/Abstract])) OR (flu vaccine[Title/Abstract])) OR (flu vaccines[Title/Abstract])) OR (influenza virus vaccine[Title/Abstract])) OR (influenza virus vaccines[Title/Abstract])) OR (universal influenza vaccine[Title/Abstract])) OR (universal influenza vaccines[Title/Abstract])) OR (universal flu vaccine[Title/Abstract])) OR (universal flu vaccines[Title/Abstract]) |
| #3 Search | #1 OR #2 |
| #4 Search | “Vaccination Coverage"[Mesh] |
| #5 Search | (((Vaccination Coverage[Title/Abstract])) OR (Vaccination Coverages[Title/Abstract])) OR (immunization coverage[Title/Abstract])) OR (immunization coverages[Title/Abstract]) |
| #6 Search | #4 OR #5 |
| #7 Search | #3 AND #6 |
