# Supplementary material for: Influenza vaccination rates among healthcare workers: a systematic review and meta-analysis investigating influencing factors
Source: Front Public Health. 2023 Nov 6;11:1295464. doi: 10.3389/fpubh.2023.1295464 (PMC10657874; doi:10.3389/fpubh.2023.1295464)
Supplement: Supplementary file 2 [file Table_2.docx]

Supplementary Table 2 Sensitivity analysis of influenza vaccination rates for each study being removed one at a time.

| Study omitted | Vaccination time | Estimate[95%*CI*] | Study omitted | Vaccination time | Estimate[95%*CI*] |
| --- | --- | --- | --- | --- | --- |
| Sheng Y.Q. | 2017 | 42.0%(35.9%, 48.1%) | Black | 2010 | 41.5%(35.4%, 47.6%) |
| Liu T. | 2017 | 41.8%(35.7%, 47.9%) | Black | 2011 | 41.5%(35.4%, 47.6%) |
| Liu T. | 2018 | 41.8%(35.7%, 47.9%) | Hussain | 2011 | 41.4%(35.3%, 47.5%) |
| Wang L.J. | 2012 | 41.8%(35.7%, 47.9%) | Hussain | 2012 | 41.4%(35.4%, 47.5%) |
| Gao M. | 2013 | 41.9%(35.9%, 48.0%) | Hussain | 2013 | 41.4%(35.4%, 47.5%) |
| Liu N. | 2018 | 41.9%(35.8%, 47.9%) | Bazán | 2010 | 41.4%(35.3%, 47.4%) |
| Yang L.X. | 2013 | 41.7%(35.6%, 47.8%) | Groenewold | 2004 | 42.0%(36.0%, 48.0%) |
| Gan Z.K. | 2018 | 41.8%(35.8%, 47.9%) | Lu J. | 2006 | 41.7%(35.6%, 47.7%) |
| Wang D.M. | 2007 | 42.0%(35.9%, 48.0%) | Madewell | 2017 | 41.5%(35.4%, 47.6%) |
| Bu L.H. | 2012 | 42.0%(35.9%, 48.1%) | Madewell | 2018 | 41.6%(35.5%, 47.7%) |
| Yang J.S. | 2016 | 42.0%(35.9%, 48.1%) | James | 2016 | 42.0%(35.9%, 48.1%) |
| Wang Z.W. | 2017 | 42.0%(35.9%, 48.1%) | Harrison | 2013 | 41.7%(35.6%, 47.8%) |
| Zhang Q.H. | 2017 | 41.9%(35.8%, 48.0%) | Murray | 1999 | 41.6%(35.6%, 47.7%) |
| Kong Q.F. | 2019 | 41.8%(35.7%, 48.0%) | Hosamirudsari | 2015 | 41.6%(35.5%, 47.6%) |
| Ma Y.Y. | 2017 | 42.0%(35.9%, 48.1%) | Alhammadi | 2013 | 41.5%(35.4%, 47.6%) |
| Gan L. | 2018 | 41.9%(35.8%, 48.0%) | Garcell | 2011 | 41.5%(35.5%, 47.6%) |
| Liu H. | 2018 | 41.9%(35.8%, 48.0%) | Garcell | 2012 | 41.5%(35.4%, 47.5%) |
| Wong N.S. | 2013 | 41.8%(35.7%, 47.9%) | Haridi | 2012 | 41.6%(35.5%, 47.7%) |
| Wong N.S. | 2014 | 41.7%(35.7%, 47.8%) | Haridi | 2013 | 41.5%(35.5%, 47.6%) |
| Wong N.S. | 2015 | 41.8%(35.7%, 47.8%) | Haridi | 2014 | 41.3%(35.3%, 47.4%) |
| Wong N.S. | 2016 | 41.7%(35.7%, 47.8%) | Rehmani | 2008 | 41.8%(35.7%, 47.8%) |
| Wong N.S. | 2017 | 41.7%(35.6%, 47.8%) | Hakim | 2018 | 41.8%(35.7%, 47.9%) |
| Hudu | 2013 | 41.6%(35.5%, 47.7%) | Tagajdid | 2011 | 41.9%(35.8%, 48.0%) |
| Kan | 2011 | 41.8%(35.7%, 47.8%) | Hajiabdolbaghi | 2019 | 41.8%(35.7%, 47.9%) |
| Yi H. | 2019 | 41.5%(35.4%, 47.6%) | Dubnov | 2004 | 41.9%(35.8%, 48.0%) |
| Yu J. | 2017 | 42.0%(35.9%, 48.1%) | Khazaeipour | 2008 | 41.5%(35.4%, 47.6%) |
| Boey | 2014 | 41.5%(35.4%, 47.6%) | Department | 2015 | 41.3%(35.3%, 47.4%) |
| Boey | 2015 | 41.6%(35.5%, 47.7%) | Waheed | 2019 | 41.9%(35.8%, 48.0%) |
| Barbadoro | 2012 | 41.9%(35.7%, 48.0%) | Khazaeipour | 2008 | 41.5%(35.4%, 47.6%) |
| Rabensteiner | 2015 | 42.0%(35.9%, 48.0%) | Jiang S.Q. | 2019 | 41.8%(35.7%, 47.9%) |
| Costantino | 2011 | 41.9%(35.9%, 48.0%) | Fan X. | 2019 | 41.9%(35.8%, 48.0%) |
| Jimenez-Garcia | 2001 | 41.9%(35.8%, 48.0%) | Yan R. | 2019 | 41.7%(35.6%, 47.7%) |
| Jimenez-Garcia | 2003 | 41.9%(35.8%, 47.9%) | Li M. | 2019 | 41.7%(35.6%, 47.8%) |
| Perbandt | 2014 | 41.9%(35.8%, 48.0%) | Li M. | 2020 | 41.6%(35.5%, 47.6%) |
| Socˇan | 2009 | 41.6%(35.5%, 47.7%) | Zhang B.L. | 2015 | 41.8%(35.7%, 47.8%) |
| Domínguez | 2011 | 41.6%(35.5%, 47.7%) | Zhang B.L. | 2016 | 41.7%(35.7%, 47.8%) |
| Hagemeister | 2012 | 41.7%(35.6%, 47.8%) | Zhang B.L. | 2017 | 41.7%(35.7%, 47.8%) |
| Hagemeister | 2013 | 41.6%(35.5%, 47.7%) | Zhang B.L. | 2018 | 42.0%(35.9%, 48.1%) |
| Hagemeister | 2014 | 41.6%(35.5%, 47.7%) | Zhang B.L. | 2019 | 41.8%(35.7%, 47.8%) |
| Jesús Castilla | 2008 | 41.5%(35.5%, 47.6%) | Wu Z.L. | 2018 | 41.9%(35.8%, 48.0%) |
| Castilla | 2009 | 41.5%(35.5%, 47.6%) | Lu L.X. | 2018 | 41.9%(35.8%, 48.0%) |
| Castilla | 2010 | 41.6%(35.5%, 47.7%) | Fan Z.L. | 2019 | 41.6%(35.5%, 47.7%) |
| Castilla | 2011 | 41.6%(35.5%, 47.7%) | Fan Z.L. | 2020 | 41.3%(35.3%, 47.4%) |
| Tanguy | 2009 | 41.9%(35.8%, 47.9%) | Lei M.L. | 2018 | 42.0%(35.9%, 48.1%) |
| Amodio | 2009 | 41.9%(35.8%, 47.9%)) | Lei M.L. | 2020 | 41.9%(35.8%, 47.9%) |
| Dorribo | 2009 | 41.6%(35.5%, 47.7%) | Ma L. | 2020 | 41.7%(35.6%, 47.8%) |
| Sánchez-Payá | 2009 | 41.8%(35.7%, 47.9%) | Ma L. | 2021 | 41.7%(35.7%, 47.8%) |
| Sánchez-Payá | 2010 | 41.8%(35.7%, 47.9%) | Papageorgiou | 2019 | 41.8%(35.7%, 47.9%) |
| Buxmann | 2016 | 41.7%(35.6%, 47.8%) | Bazán | 2016 | 41.8%(35.7%, 47.8%) |
| Domínguez | 2011 | 41.6%(35.5%, 47.7%) | Bazán | 2019 | 41.6%(35.5%, 47.7%) |
| Toledo | 2013 | 41.8%(35.8%, 47.9%) | Bazán | 2020 | 41.5%(35.4%, 47.5%) |
| Loulergue | 2006 | 41.6%(35.5%, 47.7%) | Bertoni | 2020 | 41.6%(35.5%, 47.6%) |
| Petek | 2014 | 41.9%(35.9%, 48.0%) | Bertoni | 2019 | 41.8%(35.8%, 47.9%) |
| Vírseda | 2009 | 41.6%(35.6%, 47.7%) | Bertoni | 2018 | 41.9%(35.8%, 47.9%) |
| Hämäläinen | 2018 | 41.2%(36.9%, 45.6%) | Marinos | 2020 | 41.4%(35.4%, 47.5%) |
| Hämäläinen | 2015 | 41.6%(35.5%, 47.6%) | Shi X. | 2020 | 41.7%(35.6%, 47.8%) |
| Kent | 2007 | 41.4%(35.4%, 47.5%) | Jedrzejek | 2018 | 41.6%(35.6%, 47.7%) |
| Black | 2012 | 41.5%(35.4%, 47.5%) | Jedrzejek | 2019 | 41.5%(35.5%, 47.6%) |
| Black | 2013 | 41.4%(35.4%, 47.5%) | Costantino | 2020 | 41.6%(35.5%, 47.6%) |
| Black | 2014 | 41.4%(35.3%, 47.5%) | Ogliastro | 2019 | 41.9%(35.8%, 48.0%) |
| Black | 2015 | 41.4%(35.3%, 47.5%) | Ogliastro | 2020 | 41.6%(35.5%, 47.7%) |
| Black | 2016 | 41.4%(35.3%, 47.5%) | Ogliastro | 2021 | 41.8%(35.7%, 47.9%) |
| Black | 2017 | 41.4%(35.3%, 47.5%) |  |  |  |
